# Expression and Activity of the NF-κB Subunits in Chronic Lymphocytic Leukaemia: A Role for RelB and Non-Canonical Signalling

**DOI:** 10.3390/cancers15194736

**Published:** 2023-09-26

**Authors:** Evan A. Mulligan, Susan J. Tudhope, Jill E. Hunter, Arabella E. G. Clift, Sarah L. Elliott, Geoffrey P. Summerfield, Jonathan Wallis, Chris J. Pepper, Barabara Durkacz, Stephany Veuger, Elaine Willmore

**Affiliations:** 1 Cancer Research UK Drug Discovery Unit, Newcastle University, Newcastle upon Tyne NE2 4HH, UK; 2 Department of Haematology, Queen Elizabeth Hospital, Gateshead NE9 6SX, UK; 3 Northern Centre for Cancer Care, Freeman Hospital, Newcastle upon Tyne NE7 7DN, UK; 4 Medical Research Building, Brighton and Sussex Medical School, University of Sussex, Brighton BN1 9PX, UK; 5 Department of Applied Sciences, Northumbria University, Newcastle upon Tyne NE7 7XA, UK

**Keywords:** NF-κB, CLL, RelB

## Abstract

**Simple Summary:**

Chronic lymphocytic leukaemia (CLL) patients frequently experience drug-resistance. Signalling via the transcription factor NF-κB is a major contributor to resistance due to its ability to regulate the expression of genes that confer growth and survival of CLL cells. NF-κB signalling comprises ‘canonical’ and ‘non-canonical’ pathways involving the subunits RelA and RelB, respectively. RelA has been widely investigated in resistance and outcome in CLL but RelB activity and therapeutic implications are less well understood. We sought to examine RelB expression and function using a large cohort of comprehensively annotated patient-derived CLL cells. We demonstrate that RelB activity is associated with specific subsets of patients and with poorer outcome. Use of CD40L-expressing cells to represent the in vivo microenvironment led to RelB activation and CLL cell proliferation. Strategies to develop novel agents that target non-canonical NF-kB signalling will enable patient stratification and the development of more personalized therapy for CLL patients.

**Abstract:**

Background: Canonical NF-κB signalling by p65 (RelA) confers chemo-resistance and poor survival in chronic lymphocytic leukaemia (CLL). The role of non-canonical NF-κB signalling (leading to RelB and p52 subunit activation) in CLL is less understood, but given its importance in other B-cell tumour types, we theorised that RelB and p52 may also contribute to the pathology of CLL. Methods: DNA binding activity of all five NF-kB subunits, p65, p50, RelB, p52, and c-Rel, was quantified using ELISA and correlated to ex vivo chemoresistance, CD40L-stimulated signalling (to mimic the lymph node microenvironment), and clinical data. Results: Importantly, we show for the first time that high basal levels of RelB DNA binding correlate with nuclear RelB protein expression and are associated with del(11q), ATM dysfunction, unmutated IGHV genes, and shorter survival. High levels of nuclear p65 are prevalent in del(17p) cases (including treatment-naïve patients) and also correlate with the outcome. CD40L-stimulation resulted in rapid RelB activation, phosphorylation and processing of p100, and subsequent CLL cell proliferation. Conclusions: These data highlight a role for RelB in driving CLL cell tumour growth in a subset of patients and therefore strategies designed to inhibit non-canonical NF-κB signalling represent a novel approach that will have therapeutic benefit in CLL.

## 1. Introduction

The introduction of treatment regimens including chemo-immunotherapy have significantly improved response to therapy for patients with chronic lymphocytic leukaemia (CLL); however, recent advances targeting the ability of receptor signalling to maintain cell survival, and the anti-apoptotic function of BCL2 are proving highly successful, highlighting the key role of these pathways in CLL [1]. Although many patients achieve a good response, resistance to both targeted and chemotherapeutic agents occurs, e.g., via genomic alterations [2] and a subset of patients with p53 dysfunction are chemoresistant [3]. ATM (ataxia telangiectasia mutated kinase) also plays a role in chemoresistance: it has numerous roles in DNA damage signalling, cell cycle checkpoint control, and homologous recombination repair and patients with loss of ATM (del(11q) and ATM mutation) also have a poorer survival [4].

Another factor that is strongly associated with drug resistance is the stress-inducible transcription factor NF-κB, which comprises five subunits; p65 (also termed ‘RelA’), RelB, c-Rel, p50, and p52. These subunits form homo- and heterodimers and are associated with specific pathways of activation. p65, p50, and c-Rel are stimulated by the canonical route of activation, which is mediated by the inhibitor of kappa B kinase β (IKKβ) complex. In unstimulated cells, the subunits are inactive in the cytoplasm through interaction with the inhibitor κB (IκB) proteins. Stimulation (e.g., following inflammation or DNA damage) results in IKKβ-mediated phosphorylation of IκB proteins, targeting them for proteasomal degradation, and releasing the NF-κB subunits for nuclear translocation and subsequent interaction with transcriptional co-activators to promote gene transcription [5]. As well as IKKβ, NF-κB essential modifier (NEMO) regulates DNA damage-induced canonical signalling in a process that requires ATM, resulting in activation of IKKβ [6]. Indeed, we have previously demonstrated that ATM is required for DNA damage-induced activation of p65/p50 in breast cancer cells [7], and in CLL cells ATM interacts with the TCL1 oncogene to activate NF-κB [8].

The non-canonical NF-κB signalling pathway involves NF-κB-inducing kinase (NIK)-induced activation of IKKα, which in turn promotes degradation of the p100 precursor into active p52 [6]. RelB and p52 are common heterodimeric partners and associated with a distinct set of stimuli, examples of which include the B-cell activating factor of the tumour necrosis factor family (BAFF) and the CD40 receptor [9]. Although the p52 (non-canonical) and p50 (canonical) subunits lack a transactivation domain, they cooperate with other Rel subunits or transcription factors (such as BCL3) at shared promoters, and different subunit dimer combinations may determine the specificity of the transcriptional response [5].

In multiple myeloma, mutations in NF-κB regulatory genes occur in 20% of cases. Activating mutations, leading to increased activation of the CD40 receptor or NIK, result in constitutive non-canonical NF-κB signalling [10]. In mantle cell lymphoma (MCL), resistance to ibrutinib (the BCR antagonist that targets Bruton’s tyrosine kinase) is characterised by aberrant non-canonical NF-kB signalling due to mutations in TRAF2 and BIRC3 [11]. In CLL, mutations in NOTCH1, BIRC3 [12], and MYD88 all result in altered NF-kB activity, leading to, in the case of MYD88, elevated levels of cytokines that promote CLL cell survival [13]. Importantly, a comprehensive study using whole genome sequencing confirmed that BIRC3 and TRAF3 are recurrently mutated in CLL, and that NFKB2 was a novel prognostic driver which conferred shorter time to treatment [14]. These observations confirm that defective NF-kB signalling (which may result from activating mutations in NF-kB-regulating genes), is a target for therapeutic intervention.

The activation of NF-κB impacts on many cellular processes and, in CLL, constitutive activation of the p65 subunit is associated with increased ex vivo survival of CLL cells, shorter lymphocyte doubling times, and reduced patient survival [15,16]. Inhibition of NF-κB activation has been explored in CLL cells, e.g., with parthenolide-based compounds (targeting IKK activation) and DC-1-192 (inhibiting subunit binding) which induce apoptosis and decrease NF-κB-dependent gene transcription [17,18].

Here, we examined the activity of canonical and non-canonical NF-κB signalling in 80 well-characterised CLL cases. We demonstrate that, as well as elevated activity of the canonical pathway via p65 and p50, non-canonical pathway activity via RelB is highly active in some patients, and that RelB activity is associated with shorter time to treatment and overall survival, del(11q), ATM dysfunction, and unmutated *IGHV* genes. CD40L-stimulation of CLL cells activated RelB and was accompanied by phosphorylation of p100 (the p52 precursor) prior to subsequent CLL cell proliferation. Taken together, these data identify non-canonical, as well as canonical NF-κB signalling as therapeutic targets in CLL. Importantly, this will enable patient stratification and the development of personalized therapies.

## 2. Materials and Methods

### 2.1. Patient Sample Collection and Information

This study was approved by the UK NHS Research Ethics Service, and samples were collected as part of the Newcastle Biobank (ref. 17/NE/0361) https://www.ncl.ac.uk/biobanks (accessed on 1 August 2023). Following written informed consent, patients provided peripheral blood samples, from which CLL cells were isolated using Lymphoprep (Axis Shield, Cambridgeshire, UK). Interphase FISH and a multiple ligation dependent probe assay were used to determine cytogenetic abnormalities as previously described [19]. Mann–Whitney, *t*-tests, Kaplan–Meier analysis, and other statistical analyses were performed using GraphPad PRISM software (version 9) (http://www.graphpad.com, accessed on 1 August 2023).

### 2.2. NF-κB DNA Binding Activity and Expression

NF-κB activity was quantified in nuclear extracts using the Trans-AM NF-κB family Transcription Factor Assay ELISA Kit (Active Motif, Rixensart, Belgium), and using Western blotting with specific antibodies. Nuclear extracts were prepared from CLL cells using a NE-PER kit (Thermo-Scientific, Rockford, IL, USA). The ELISA has been successfully validated by us and others, showing lack of DNA binding in NF-κB null cells, and having negligible inter-assay variability [20,21]. This approach is optimal for measurement of heterogeneity of NF-κB subunits in a patient cohort, as, unlike the EMSA (which also measures DNA binding capacity) the ELISA is quantitative and high-throughput. The assay measures DNA binding capacity of active (nuclear) NF-κB to an NF-κB consensus sequence using specific antibodies to detect the individual subunits. A quantitative measurement of NF-κB binding was achieved by use of a standard calibration curve (MDA-MB-231 nuclear extracts), and results for CLL nuclear extracts (shown as arbitrary units, AU) were calculated per µg of nuclear extract from the standard curve (which was run in each assay). The MDA-MB-231 extracts were used as controls for both western blotting and ELISA because they consistently expressed a robust increase in all 5 subunits. Proprietary mutant and wild type NF-κB consensus sequence oligonucleotides were used to validate NF-κB subunit binding.

### 2.3. Western Blotting

Nuclear extracts (prepared as described above) were run on tris-acetate, 3–8% (*v*/*v*), denaturing polyacrylamide gradient gels (Bio-Rad, Herts, UK). Samples were transferred onto Hybond C extra membranes (Amersham, Bucks, UK) and probed with antibodies for p52 (Millipore, Watford, UK, Rel B (Santa Cruz, Insight Biotechnology, Middlesex, UK), PARP1 (Santa Cruz), Actin (Calbiochem, Merck, San Diego, CA, USA), p100, and phospho-p100 (both from Cell Signalling, Danvers, MA, USA). Horseradish peroxidase-conjugated secondary antibodies (Dako, Ely, UK) and ECL (GE Healthcare, London, UK) were used for detection. Original, uncropped Western blot membranes can be found in Supplementary File S1.

### 2.4. Cytotoxicity Assays in CLL Cells

For cytotoxicity studies, freshly isolated CLL cells were cultured in RPMI 1640 medium supplemented with 10% (*v*/*v*) foetal bovine serum, penicillin (50 U/mL), and streptomycin (50 µg/mL). The XTT assay (Roche Diagnostics, Sussex, UK) was used as previously described [19] to derive LC50 values (concentration that reduced viability to 50% of untreated controls) following treatment with fludarabine (Sigma, Poole, Dorset, UK).

### 2.5. Co-Culture with CD40L-Expressing Cells

CLL cell co-culture on CD40L-expressing fibroblasts was performed as described previously [22]. The CD40L status of the fibroblasts was regularly confirmed by CD154 surface expression (flow cytometry, not shown). CLL cells were stained with CFSE (carboxyfluorescein diacetate, succinimidyl ester) which forms fluorescent protein adducts that can be traced in daughter cells following cell division. These cells were co-cultured (+10 ng/mL Interleukin 4) on CD40L-expressing fibroblast cells (or on non-CD40L-expressing (NTL) cells) that had been growth-arrested (with 75 Gy ionising radiation). Cell samples were removed daily to prepare nuclear and cytoplasmic cell extracts. In parallel samples, quantification of CFSE (in CD19+ve CLL cells) using flow cytometry was used to show CLL cell proliferation (seen by sub-peaks of CFSE fluorescence) that continued throughout the period studied. 

## 3. Results

### 3.1. NF-κB Subunit Quantification in CLL Patient Samples

**Figure 1:**
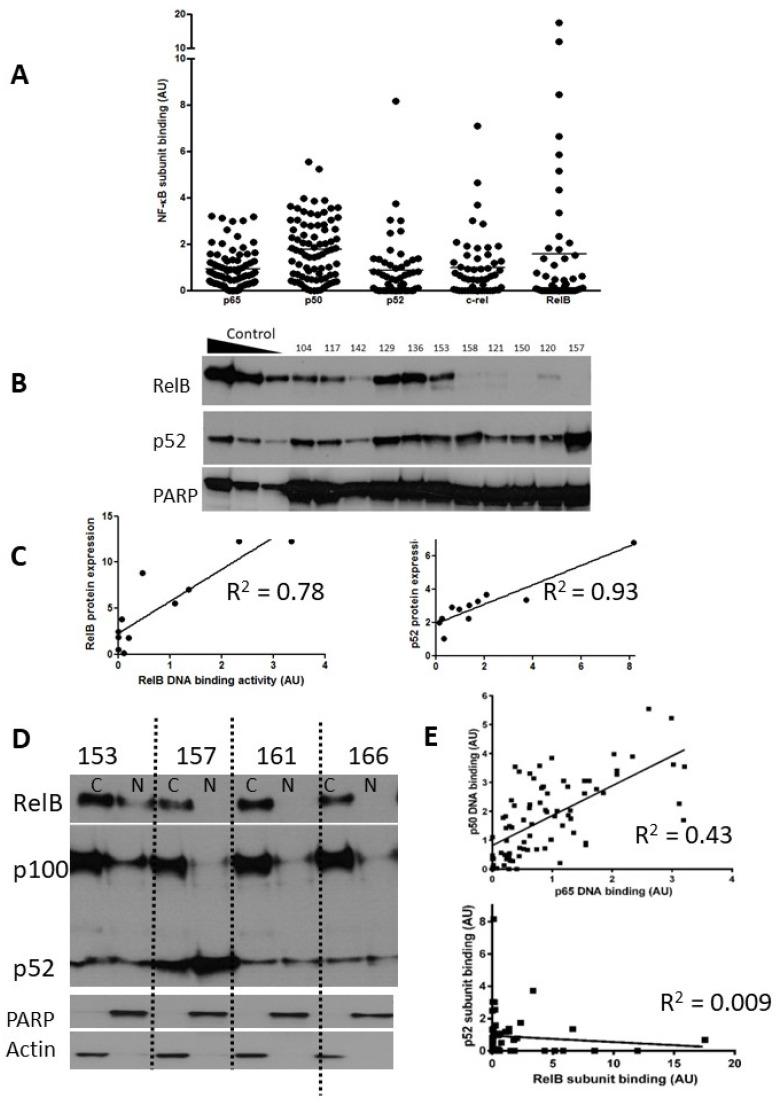
Expression of NF-kB subunits in CLL. Nuclear extracts prepared from selected CLL cases were examined using both ELISA and Western blotting. The scattergram (**A**) shows DNA binding levels of NF-κB p65 and p50 subunits (n = 80) and p52, c-Rel, and RelB subunits (n = 52) as measured using ELISA (arbitrary units AU, see methods). (**B**) Western blotting confirmed the heterogeneity in RelB and p52 expression in CLL cases. Nuclear extracts prepared from IR-treated MDA-MB-231 cells containing increasing amounts of protein (5, 10, 20 μg) were used as controls for increasing subunit expression and PARP was used as a loading control. (**C**) For both RelB and p52 there was a strong correlation between DNA binding activity and protein expression. (**D**) Expression of RelB, p100, and p52 in cytoplasmic and nuclear fractions prepared from cells from 4 CLL cases. (**E**) Correlation between p65 and p50 subunits in CLL samples (n = 80), and lack of correlation between p52 and RelB (n = 52).

We measured constitutive DNA binding levels of all five NF-κB subunits (n = 80 patients for p65 and p50, and of these 80, n = 52 patients for RelB, p52, and c-Rel). All experiments were performed on the same group of patients, described in Supplementary Table S1, which details the results for DNA binding, MLPA/cytogenetic abnormalities, IGHV mutation status, fludarabine LC_50_, and associated clinical information (Binet stage, treatment, sex, age). Figure 1A shows the NF-κB subunit DNA binding data in a scattergram with the mean DNA binding activity of each subunit shown as a solid line.

p65 and p50 were present in the majority of cases, and there was less heterogeneity in comparison to the levels of the other subunits (Figure 1A). Notably, RelB showed levels ranging from undetectable to levels that were 8–11-fold higher than the mean. Since expression and activity of p65 (RelA) in CLL is well documented, we focused on expression of RelB and its common heterodimeric partner, p52. Induction of RelB–p52 complexes is known to occur following activation of non-canonical NF-κB signalling, a pathway whose function is not yet fully understood in CLL. In order to confirm that high RelB DNA binding activity (as determined using ELISA) was reflective of elevated nuclear RelB protein expression, nuclear extracts were run in parallel experiments for detection using both ELISA and Western blotting. Figure 1B shows that that although RelB and p52 expression were variable between patient samples, they were often expressed at high levels. There was a strong correlation between RelB and p52 subunit DNA binding activity and their respective protein expression (R = 0.78 and R^2^ = 0.93, respectively; Figure 1C). Interestingly, RelB was present in the cytoplasmic fraction of the majority of CLL cases examined (as exemplified in 3/4 cases shown in Figure 1D) but was only constitutively activated in the nucleus in a subset of cases (illustrated in Figure 1B). Similarly, p100 was often present and although processing to (nuclear) p52 was variable (Figure 1D) nuclear p52 was frequently observed (Figure 1B). We examined the relationship between the DNA binding levels of the subunits (Figure 1E) and found a strong correlation between the canonical subunits p65 and p50 (n = 80, *p* < 0.001), but not between p52 and RelB (n = 52, *p* = 0.5).

### 3.2. Unmutated IGHV and High-Risk Cytogenetic Abnormalities Are Associated with Increased NF-κB Activation

**Figure 2:**
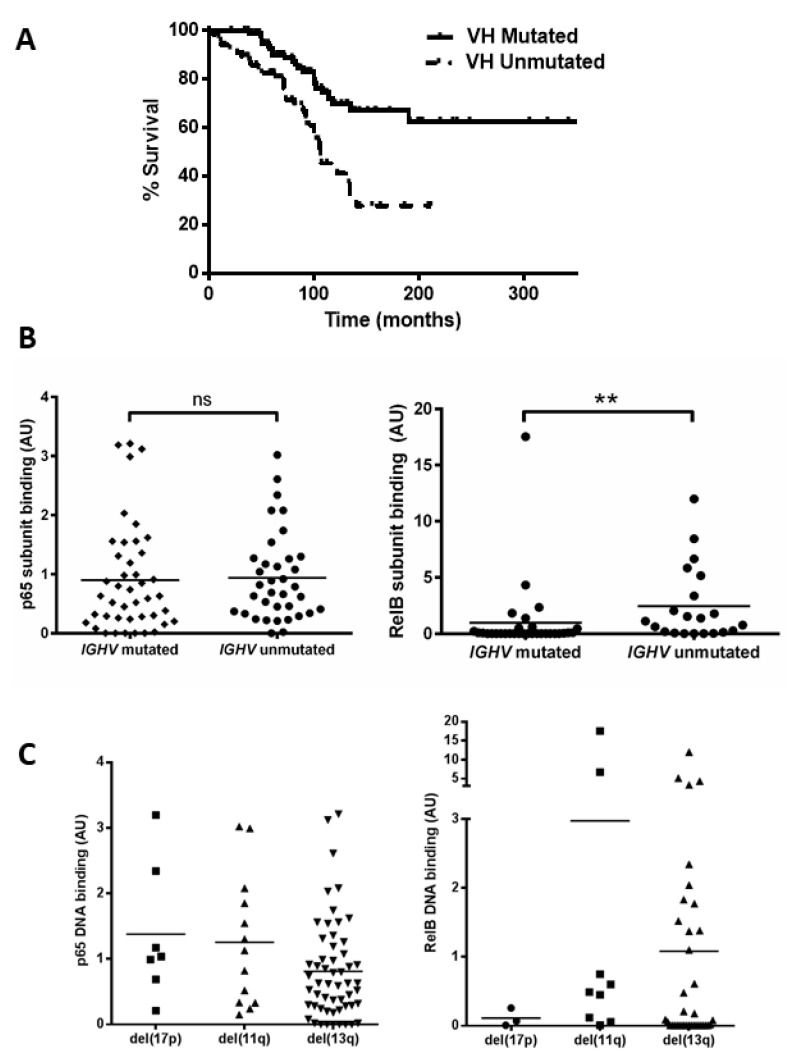
Unmutated IGHV is associated with increased RelB activation. (**A**) Kaplan–Meier analysis shows the expected shorter survival for patients with unmutated *IGHV* genes (hazard ratio 2.7). A total of 52 cases were separated into two groups according to whether they had mutated or unmutated *IGHV* genes, and the p65 and RelB DNA binding level of both groups is shown (**B**). DNA binding levels of p65 and RelB separated according to cytogenetic abnormalities: del(17p), del(11q), and del(13q) (**C**). ** denotes *p* value of <0.01 (ns = not significant).

Mutational status of the immunoglobulin heavy chain variable (*IGHV*) gene was examined (n = 168, which includes the 80 cases examined for NF-kB activity) to confirm that our cohort displayed the expected outcome and to determine whether there was any relationship between NF-κB levels and *IGHV* status. Patients with unmutated *IGHV* genes showed a significant reduction in median overall survival (OS) (Figure 2A, *p* = 0.0002, hazard ratio (HR) = 2.7). We then analysed cases for which both *IGHV* status and subunit DNA binding were known, and separated cases according to IGHV mutational status (Figure 2B). There was no significant difference between the *IGHV* mutated and unmutated groups for the p65 subunit (*p* = 0.4); however, RelB levels were higher in samples with unmutated *IGHV* genes (*p* = 0.001, despite the presence of one sample (CLL 104) that was an outlier with a very high RelB level in the ‘mutated *IGHV*’ group). This sample was subsequently found to lack functional ATM.

We then examined the DNA binding activity of the p65 and RelB subunits among patients in the different cytogenetic groups. Figure 2C shows variation in p65 levels among patients, e.g., with means of 1.4 and 0.8 for del(17p) and del(13q), respectively. Interestingly the two cases with the highest p65 levels (CLL 16 and CLL 61) had received treatment at the time of sample collection. There was also a wide variation in RelB levels, e.g., means of 0.1 versus 1.0 for del(17p) cases compared to del(13q) cases, respectively, but there was a small number of cases and this was not significant. RelB levels appeared higher in del(11q) cases but this was not significant (mean 3.0 versus 1.0 for del(13q) cases, *p* = 0.15). The del(11q) group included two outliers, with very high levels (CLL 104 and CLL 117) of RelB, and both cases were untreated at the time of sample collection and analyses, but were also subsequently found to have lack of function of ATM. We also analysed subunit activity to compare levels in patients that were treatment-naïve versus those that had received treatment (at the time of sample collection). Although there were some trends for higher levels of, e.g., RelA and RelB in treated cases, none of the comparisons were statistically significant (Supplementary Figure S1).

### 3.3. Ex Vivo Chemoresistance Is Significantly Associated with High p65 and p50 Levels but Not p52 or RelB

**Figure 3:**
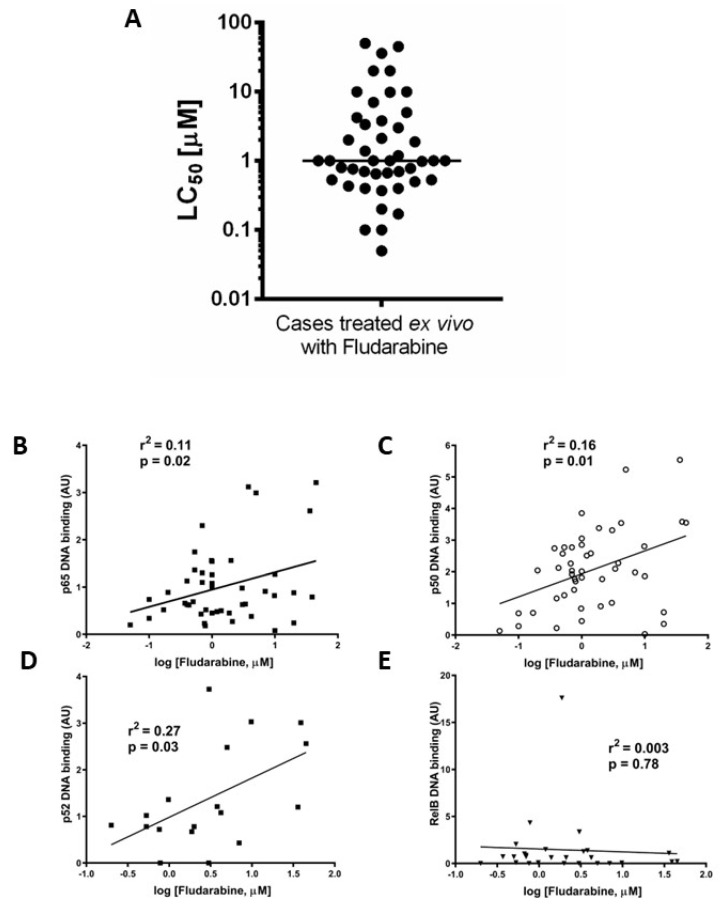
Ex vivo chemoresistance is significantly associated with high p65, p50, and p52 activity but not RelB. Cells from CLL cases were treated with fludarabine for 48 h and viability was assessed using the XTT assay. The LC_50_ values (concentration of drug that reduced viability to 50% of solvent controls) were calculated and are summarised in (**A**). LC_50_ values from fludarabine-treated cells were analysed to study relationships with p65 (**B**), p50 (**C**), p52 (**D**), and RelB (**E**).

We previously reported that ex vivo chemoresistance in CLL correlated with known markers of poor prognosis including p53 dysfunction [19]. We used a similar approach here to determine LC_50_ values for fludarabine (48 h, XTT assay) to explore the relationship between the nuclear DNA binding activity of NF-κB subunits and ex vivo drug sensitivity. Figure 3A shows that the median LC_50_ value (for cases in which both ex vivo drug sensitivity and NF-κB DNA binding activity were performed, n = 45) was 1 µM. Increased p65 (Figure 3B) and p50 (Figure 3C) subunit DNA binding was associated with resistance to fludarabine (*p* = 0.02, R^2^= 0.11 and *p* = 0.01, R^2^= 0.16, respectively, n = 38). There was a better correlation between ex vivo chemoresistance and activity of p52 compared to p65 or p50 (Figure 3D, *p* = 0.03, R^2^= 0.27) and no correlation for RelB (Figure 3E, *p* = 0.78, R^2^= 0.003).

### 3.4. Increased Activity of RelB in ATM Dysfunctional Cases

Since the data in Figure 1A (showing RelB activity for all cases) and Figure 2B (showing RelB activity in patients with unmutated *IGHV*) indicated that the RelB subunit was constitutively high in some patients, we analysed this further. For 20 of the CLL cases examined we had previously tested the functional status of ATM. Functional ATM is defined as having the ability to phosphorylate ATM ser1981 (and SMC1 at ser966) after treatment with ionising radiation, as described in [4], whereas dysfunctional ATM lacks the ability to phosphorylate at these sites.

**Figure 4:**
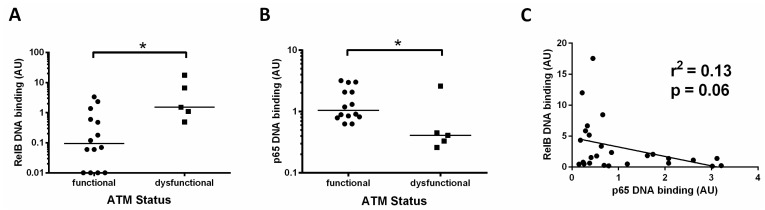
Increased activity of RelB in ATM dysfunctional cases. (**A**) Cases with ATM dysfunction tended to have higher RelB DNA binding levels than those with functional ATM; (**B**) ATM dysfunctional cases had significantly lower levels of p65; (**C**) RelB and p65 DNA binding levels showed an inverse relationship, suggesting that high levels of the RelB subunit tend to be associated with low levels of the p65 subunit. * denotes *p* value of < 0.05.

We found that patients with dysfunctional ATM had a higher level of the RelB subunit compared to cases with functional ATM (Figure 4A, median RelB levels were 0.09 versus 1.5, respectively, *p* = 0.01). By contrast, p65 levels in this subset of patients were significantly lower (median 0.4 versus 1.1, *p* = 0.02) in those cases with ATM dysfunction (Figure 4B). These data suggest a reliance on the RelB subunit in patients with loss of function of ATM. Other cases also had high RelB levels, and these were found to be predominantly cases with unmutated *IGHV* genes (Figure 2B). To further explore the relationship between canonical and non-canonical signalling, we examined p65 levels and RelB DNA binding levels and found an inverse trend, in that RelB levels were higher in cases with low p65 (*p* = 0.06, R^2^ = 0.13, Figure 4C).

### 3.5. RelB and p52 Expression Is Increased Following CD40L-Stimulated Proliferation in CLL

As well as canonical NF-κB pathway signalling, the non-canonical NF-κB pathway is known to play a role in receptor signalling [9]. We used fibroblasts that stably express CD40L in a co-culture system to more closely model the in vivo microenvironment encountered by CLL cells in the lymphoid tissues. We subsequently monitored the expression of the non-canonical subunits and assessed the proliferation of CLL cells using CFSE labelling.

**Figure 5:**
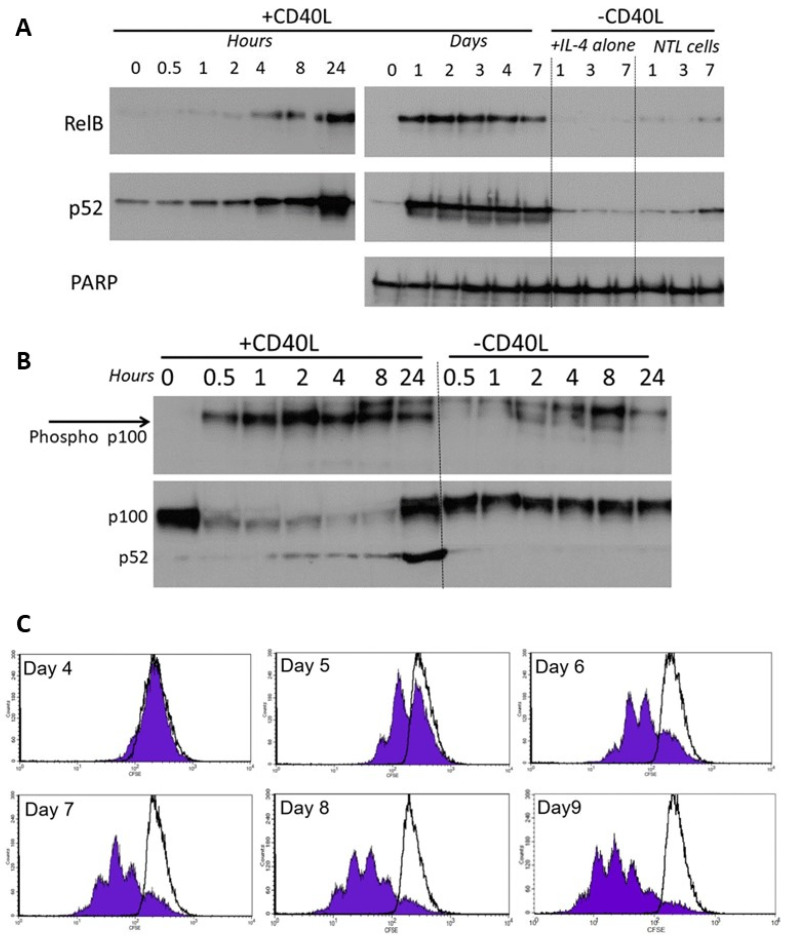
RelB and p52 are activated following CD40L-induced stimulation. (**A**) Nuclear extracts were prepared from CLL cells at specific times following co-culture on CD40L-expressing cells (n = 6, representative example shown). CLL cells with IL-4 alone, or cells lacking CD40L expression were used as controls. (**B**) Phosphorylation of cytoplasmic p100 was measured over 0.5 to 24 h after CD40 stimulation (and compared to the control (“—CD40L” conditions). (**C**) CLL cells were stained with CFSE and co-cultured (+10 ng/mL interleukin 4) on CD40L-expressing fibroblast cells or on the non-CD40L-expressing (NTL) cells (both of which had been growth-arrested with 75 Gy IR). Cell samples were removed daily and quantification of CFSE in CD19+ve CLL cells (flow cytometry), shows CLL cell proliferation (seen by sub-peaks of CFSE fluorescence) that continued throughout the period studied. CLL cells cultured on the non-CD40L-expressing cells (shown by overlaying black lines) have no CFSE sub-peaks (histograms from one case representative of 5 others).

CLL cells co-cultured on the growth-arrested CD40L-expressing fibroblasts showed a rapid increase in activation of RelB and p52 which occurred within 4 h, peaked at 24 h (Figure 5A, left panel), and persisted throughout the 7 day co-culture period (Figure 5A, right panel). Cells with IL-4 alone, or without CD40L stimulation showed little or no activation. The activation of RelB and p52 was accompanied by phosphorylation of cytoplasmic p100 (<30 min, Figure 5B), and processing of p100 to p52 consistent with corresponding activation of non-canonical NF-κB pathway signalling. There was no significant phosphorylation of p100 (nor any change in p100 processing) in cells without CD40L stimulation. CD40L-stimulated non-canonical signalling resulted in the induction of CLL cell proliferation (CFSE staining of CD19+ cells, Figure 5C).

### 3.6. High Levels of RelB Activity Are Associated with Shorter Time to Treatment and Survival in CLL

**Figure 6:**
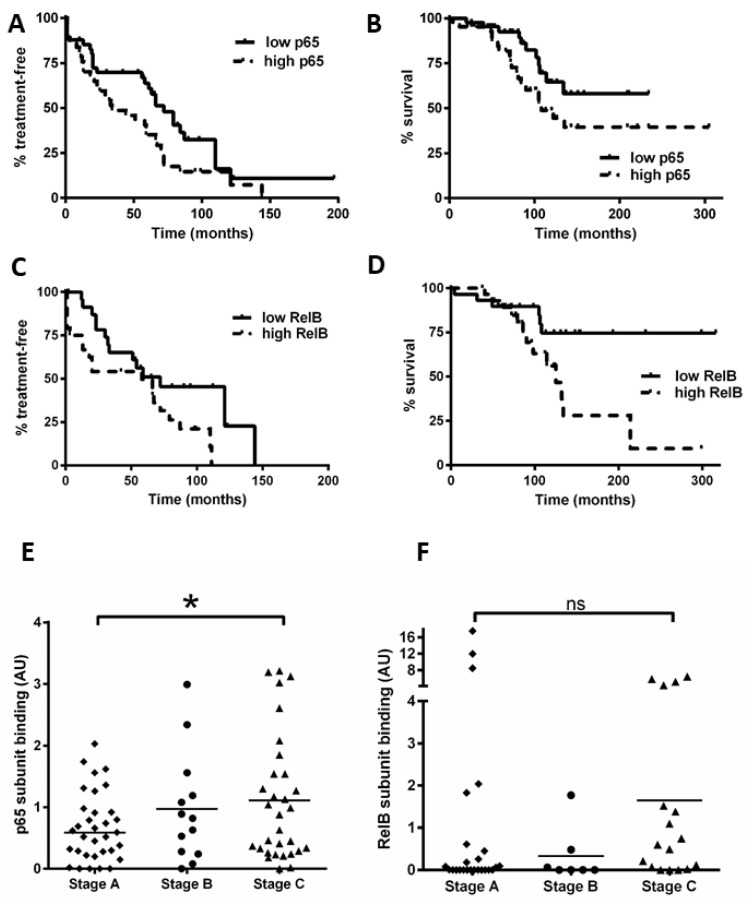
High levels of nuclear RelB activity are associated with shorter time to treatment and poorer survival in CLL. Kaplan–Meier analysis was used to examine p65 and RelB activity in relation to time from diagnosis to first treatment and overall survival (OS); cases were stratified according to whether their DNA binding levels for p65 were higher or lower than the median level. (**A**) p65 in relation to time to treatment or to OS (**B**). RelB subunit DNA binding activity in relation to (**C**) TTT or (**D**) OS. Cases were then segregated according to the Binet stage at the time of sample collection, and p65 DNA binding activity for Binet B and C cases (n = 47) was compared to that for stage A cases (n = 33) (**E**). Similarly, RelB was compared according to Binet stage (**F**) (solid line on scatter plot shows median value). * denotes a *p* value of <0.05. (ns = not significant).

Using the data obtained for Figure 1, we separated patients into groups with NF-κB subunit activity either above or below the median value. In keeping with previous studies [16], we found that a high activity of p65 was associated with a shorter time to first treatment (TTT, *p* = 0.04) and OS (*p* = 0.04; Figure 6A,B). Importantly, we show for the first time that cases with high RelB activity are associated with poorer outcome: patients with high RelB activity had a shorter TTT (Figure 6C, *p* = 0.04) and OS (Figure 6D, *p* = 0.01).

We also examined the Binet stage (at the time of sample collection) and NF-κB subunit DNA binding (n = 80 for p65, n = 52 for RelB). Figure 6E shows that Binet stage C patients had a higher level of p65 activity compared to stage A patients (*p* = 0.03). Intriguingly, Binet stage C patients tended to have a higher level of RelB activity compared to stage A patients (Figure 6F), but this did not quite reach significance (*p* = 0.06), likely due to three Binet stage A cases that had high RelB activity (including one with ATM loss). Taken together, these analyses suggest that RelB activity plays a role in disease progression and these data merit further study on an independent cohort.

## 4. Discussion

Our analysis of NF-κB subunit activity in CLL demonstrates that, as well as the p65 subunit, which has previously been demonstrated to define outcome [16], other subunits, including RelB, can be highly expressed in CLL. Previous studies have demonstrated that this is in contrast to normal, healthy B and T lymphocytes which have little or no detectable NF-kB subunits [16]. The strong correlation between the activity of p65 and p50 highlights the importance of canonical NF-κB activation in CLL. Expression and activity of p52 and RelB were highly variable and did not always correlate with one another. This discrepancy implies the presence of distinct NF-κB complexes in different CLL cases, and, coupled with our observation that RelB and p52 are rapidly and continuously activated during CD40L-stimulated proliferation, suggests that as well as canonical signalling, non-canonical NF-κB signalling contributes to CLL cell survival and proliferation. In agreement with our observations, an independent report [23] shows that RelB is present in CLL cells, and important in the context of proteasome inhibitor-induced sensitivity.

We previously measured the ex vivo response of CLL cells to fludarabine and demonstrated that chemoresistance correlated with expression of poor prognosis markers [19]. Here, we found that patients with high p50 or p65 DNA binding activity were more resistant to ex vivo treatment with fludarabine, in agreement with a previous study [16], suggesting that drug resistance is linked to increased activity of canonical NF-κB signalling. Since the expression of Rel B, p52, and c-Rel subunits were not strongly associated, it is feasible that non-canonical NF-κB signalling may not play a major role in ex vivo chemoresistance (as previously reported [23]) but is instead important in mediating other key functions such as cell proliferation. Recent reports demonstrate that recurrent mutations in *NFKB2* are drivers for CLL progression [13,14], and in the case of MCL, resistance to ibrutinib is defined by aberrant non-canonical NF-κB signalling due to mutations in *TRAF2* or *BIRC3* [11]. The trend for patients with del(17p) to have higher canonical NF-κB activity suggests that in this group of patients (known to be refractory to the effects of DNA damaging drugs), high activity of NF-κB may contribute to chemoresistance.

The importance of our novel observation that high RelB levels occur in a subset of CLL patients is extended by the observation that high RelB levels were associated with a shorter time to treatment as well as a shorter median OS in CLL. This association of activated non-canonical NF-kB signalling with poor outcome is corroborated by key survival analyses from a multicentre study, which showed that CLL patients with *BIRC3* mutations (and therefore increased non-canonical NF-kB signalling), represent a group of patients with shorter progression-free survival [12]. Indeed, future studies should examine RelB activity in CLL in the context of the mutational status of genes that are known to play a role in this signalling pathway (e.g., *BIRC3*, *NFKBIE* [13,14]).

Interestingly, we found little evidence for a major role for c-Rel. High levels of c-Rel did not affect outcome or display significant differences in terms of prognostic markers. There was also no strong relationship between c-Rel DNA binding and p50 or p65 DNA binding.

Importantly, our data from a subset of cases with ATM dysfunction showed increased DNA binding of the RelB subunit, with negligible activity in CLL cells from patients with functional ATM. These data suggest that p52/RelB heterodimers may be associated with ATM dysfunction, while alternative p52 complexes (e.g., homodimers or heterodimers with p65 or c-Rel) may be present in other contexts. Since ATM function is required for canonical pathway activation, via NEMO (NF-κB essential modifier) [6], loss of functional ATM may divert signalling towards the non-canonical route of activation and a reliance on RelB (since the p52 subunit lacks a transactivation domain). This intriguing possibility is supported by the observation that p65 levels are lower in ATM dysfunctional patients (Figure 4) and merits further study, since it validates the concept of targeting ATM-activated NF-κB. Indeed, our study in breast cancer cells [7] and a report on high-risk myelodysplastic syndrome and acute myeloid leukaemia [24] confirm that targeting ATM abrogates canonical NF-κB signalling.

We also show here that cases with unmutated *IGHV* had a higher level of RelB DNA binding activity than cases with mutated *IGHV* (Figure 2) and hypothesise that RelB function may be important in this CLL subtype, which is known to have more proficient signalling via the BCR and is reliant on pro-survival signals from the microenvironment [25]. Another study detected RelB in CLL cases with unmutated IGHV, and RelB DNA binding activity was shown to decrease with time in culture [26]. However, those cells were not co-cultured with stromal cell or CD40L-expressing cells, underlining the concept that RelB activity (and therefore non-canonical NF-κB signalling) may result from signalling in the CLL cell microenvironment.

Moreover, in CLL cells stimulated to proliferate using CD40L-transfected cells, the rapid activation of RelB and p52 (which persisted throughout the co-culture period) confirms that CD40L-mediated stimulation of CLL cells is concomitant with non-canonical NF-κB pathway signalling. Previous studies have shown that canonical NF-κB signalling and non-canonical signalling occur following CD40L-stimulation [9]. Importantly, our observation that RelB and p52 activity was accompanied by phosphorylation of p100 (within 30 min of co-culture, Figure 5C) suggests that phosphorylation by IKKα, which is known to direct processing of p100 to the active p52, is a key event in this cascade.

## 5. Conclusions

Our work highlights a novel expression pattern for the RelB subunit. We propose that targeting non-canonical NF-κB signalling is an attractive therapeutic option in CLL, particularly since its activation following CD40L-mediated signalling demonstrates its important role during the onset of CLL cell proliferation. Indeed, this concept is supported by our recent studies demonstrating that CLL cells are sensitive to prototype NIK inhibitors [27,28]. In addition to our observations, RelB activation was found to occur in a subset of poor prognosis diffuse large B-cell lymphoma patients [29]; studies in Hodgkin lymphoma (HL) confirmed that knockdown or chemical inhibition of RelB resulted in a dramatic loss of viability of HL cell lines [30] and increased non-canonical NF-κB signalling occured in ibrutinib-resistant MCL cases [11]. Furthermore, NIK, which drives IKKα-mediated non-canonical signalling, was highly expressed in HL cell lines and primary biopsies [30], indicating a mechanism by which enhanced non-canonical signalling leads to increased nuclear RelB, which in turn facilitates cell survival.

Our results strongly suggest that future studies should determine the role of RelB in identifying CLL patients that would be sensitive to inhibition of non-canonical NF-κB signalling as a means to developing personalized therapies.

## Data Availability

The data presented in this study are available in the supplementary material.

## Supplementary Materials

The following supporting information can be downloaded at: https://www.mdpi.com/article/10.3390/cancers15194736/s1, File S1: Original, uncropped Western blot membranes; Figure S1: NF-κB subunit levels in treatment-naïve versus treated cases; Table S1: NF-κB subunit levels in cases analysed and associated clinical information.
