# Supplementary material for: Expression and Activity of the NF-κB Subunits in Chronic Lymphocytic Leukaemia: A Role for RelB and Non-Canonical Signalling
Source: Cancers (Basel). 2023 Sep 26;15(19):4736. doi: 10.3390/cancers15194736 (PMC10571822; doi:10.3390/cancers15194736)
Supplement: Supplementary file 1 [file cancers-15-04736-s001.zip › cancers-2601618-supplementary.pdf]

CLL ID	cytogenetic abnormalities	Binet Stage	*IGHV* status	Treatment	p65	p50	p52	c-rel	RelB	Age	sex	LC_{50}
10	del(13q)	A	U	none	0.92	2.62				78	F	
12	del(13q)	C	U	CLB, FC	0.46	0.73				57	M	
13	del(17p)	C	U	none	1.17	1.51				58	M	
16	del(17p)	C	M	CLB	3.19	1.69	0.52	0.03	0.07	90	M	
18	del(11q)	C	U	none	1.3	2.03				72	M	0.7
21	del(13q)	C	M	F	0.63	1.16				79	F	0.4
24	del(13q)	A	U	none	1.26	2.85				79	F	1
25	del(13q)	C	M	CLB	1.54	3.05				69	M	1
28	del(13q)	A	U	none	0.62	2.12				74	F	0.43
33	del(13q)	B	U	none	1.08	2.01				38	M	1
35	del(17p)	C	M	none	0.99	3.85				75	F	1
38	del(13q)	C	U	CLB, FC, R	0.45	0.44				58	M	1
40	del(11q)	C	U	none	0.34	0.28				57	M	0.1
43	del(13q)	A	M	CLB	0.2	0.13				66	F	0.05
46	none	A	M	none	1.31	1.75	0.58	0	0.06	67	F	
47	del(11q)	A	M	none	0.52	1.7				78	M	0.8
48	del(11q)	C	U	CLB, FC	1.13	0.22				61	M	0.4
50	del(13q)	B	U	none	0	1.11				58	M	
61	del(17p)	B	U	F	2.34	3.89				60	F	
63	del(17p)	C	U	F	1.27	1.86				60	F	10
64	del(11q)	C	M	CLB	1.85	2.63				73	M	
67	del(11q)	C	U	CLB, F, FCR	1.54	2.61				58	M	
69	del(13q)	C	M	CLB, C, F	0.88	0.72				68	F	20
70	del(13q)	B	M	CLB	0.08	0.03				81	F	10
71	none	B	M	CLB	0.24	0.35				75	M	20
72	del(13q)	A	M	none	0.91	1.98	0.43	1.17	0	59	M	7
73	del(13q)	B		CLB	0.28	0.39				75	M	
75	del(13q)	C	U	CLB, FC	0.02	0.14	0.27	0.03	0.02	81	F	
76	del(17p)	C	U	none	1.04	1.82				62	M	1
84	del(13q)	A	M	CLB	0.52	0.7				86	M	0.2
85	del(13q)	A	M	CLB	0.74	0.69				82	M	0.1
89	del(13q)	A	M	none	0	0				60	M	
90	del(13q)	A	M	none	0.98	1.02	0	0	0	60	M	3
91	del(13q)	A	M	none	0.02	0.37	0	0.12	0	55	F	
93	del(13q)	A	M	none	0	0.47	0	0	0	51	M	
96	del(13q)	A	M	none	0.3	0.3	0.15	0	0	81	F	
97	del(13q)	A	M	none	0.59	0.67	0	0	0	71	F	
101	12+	A	U	none	0.66	1.56	0	0.45	8.45	60	M	
102	del(13q)	A	U	none	0.22	1.82	0	0.6	12	67	M	
103	del(13q)	C	U	CLB, F, CHOP	0.41	0	0	0.63	1.52	59	M	
104	del(11q)	A	M	none	0.45	3.38	0.67	1.36	17.6	82	M	1.9
105	none	A	U	C	1.74	2.77	0.78	1.91	2.04	66	F	0.5
106	del(13q)	C	M	F, FC	0.18	1.77	0	0.15	4.33	84	F	0.8

107	del(13q)	C	M	CLB, FC	0	0.42	0.23	1.02	0	73	M	
108	del(11q)	A	M	none	0.15	0.57	0	0.53	0.45	67	M	
109	del(13q)	A	M	none	0	0.04	0	0.47	0	71	M	
110	none	A	M	CLB	1.62	3.05	0	1.83	1.83	87	M	
111	none	B	M	none	0.63	2.23	0.11	1.54	0	71	F	
112	del(13q)	A	M	FC	0.32	2.38	0.07	2.08	0	70	M	
113	none	C	U	CLB, F	0.37	2.19	0	0.74	5.16	78	M	
114	del(17p)	C	U	none	0.21	0.49	1.31	0.48	0	68	M	
115	12+	C	U	CLB, FC	0.29	1.49	0	1.5	5.85	70	M	
116	del(11q)	C	M	none	0.26	0.48	0.11	1.9	0.49	82	M	
117	del(11q)	C	U	none	0.33	1.44	1.35	3.01	6.65	78	F	
118	none	A	M	none	0.28	0	0	0.86	0	61	M	
119	del(13q)	B	U	CLB	0.53	0.94	0.67	4.64	1.77	62	F	
120	del(13q)	B	U	CLB	0.89	2.04	0.81	0	0	43	M	0.2
121	12+	C	U	none	0.23	1.43	0.72	0	0	33	M	0.8
124	none	A	M	none	1.36	1.26	1.02	0.95	0.09	57	M	0.5
125	del(13q)	A	M	CLB	1.56	0.84	1.36	0	0.08	73	F	1
127	del(13q)	B	M	CLB	1.56	0.91	0.78	0	0	70	M	2
129	none	C	U	none	0.63	3.31	3.73	1.84	3.36	52	M	
132	del(11q)	B	M	none	2.99	5.23	2.48	0	0	62	M	5
136	del(13q)	C	M	CLB	0.85	2.85	1.73	2.86	2.34	73	M	
142	del(13q)	C	U	CLB, FC, CHOP	2.61	5.54	1.18	1.2	1.1	60	M	36
143	del(11q)	C	U	FC	0.24	0.99	0.5	0.04	0.75	55	M	
144	del(13q)	A	M	CLB	0	0.35	0.02	0.01	0.01	74	F	
148	none	C	M	CLB, FC	3.12	2.27	1.21	7.1	1.38	65	M	3.8
150	none	A	M	CLB	2.03	3.98	0.27	0	0	71	F	
153	del(13q)	B	U		2.08	3.4	1.37	1.26	1.37	63	M	
157	del(11q)	C	U	none	3.02	3.62	8.16	3.68	0.12	62	M	
158	del(11q)	C	U	CLB, FCR	2.08	3.27	0	0	0.6	62	M	
160	del(13q)	A	M	none	0.8	2.3	0.8	0.24	0	72	F	
161	del(17p)	A	U	none	0.69	3.14	1.57	0.46	0.26	54	M	
162	del(13q)	B	M	F, FCR	1.19	3.05	0.96	0.9	0.48	57	M	
164	del(13q)	A	M	none	0.29	0.54	0	0	0	53	F	
165	del(13q)	A	M	none	0.38	3.54	1.08	0.92	0.61	66	M	4.2
166	none	A	U	none	0.79	3.58	3.01	0.67	0.18	77	M	50
167	del(11q)	B	U	none	0.82	2.8	3.03	0.91	0.06	48	M	9.8
168	del(13q)	C	M	none	3.21	3.55	2.56	0.49	0.21	55	F	45

Supplementary Table S1. NF-kB subunit levels in cases analysed and associated clinical information.

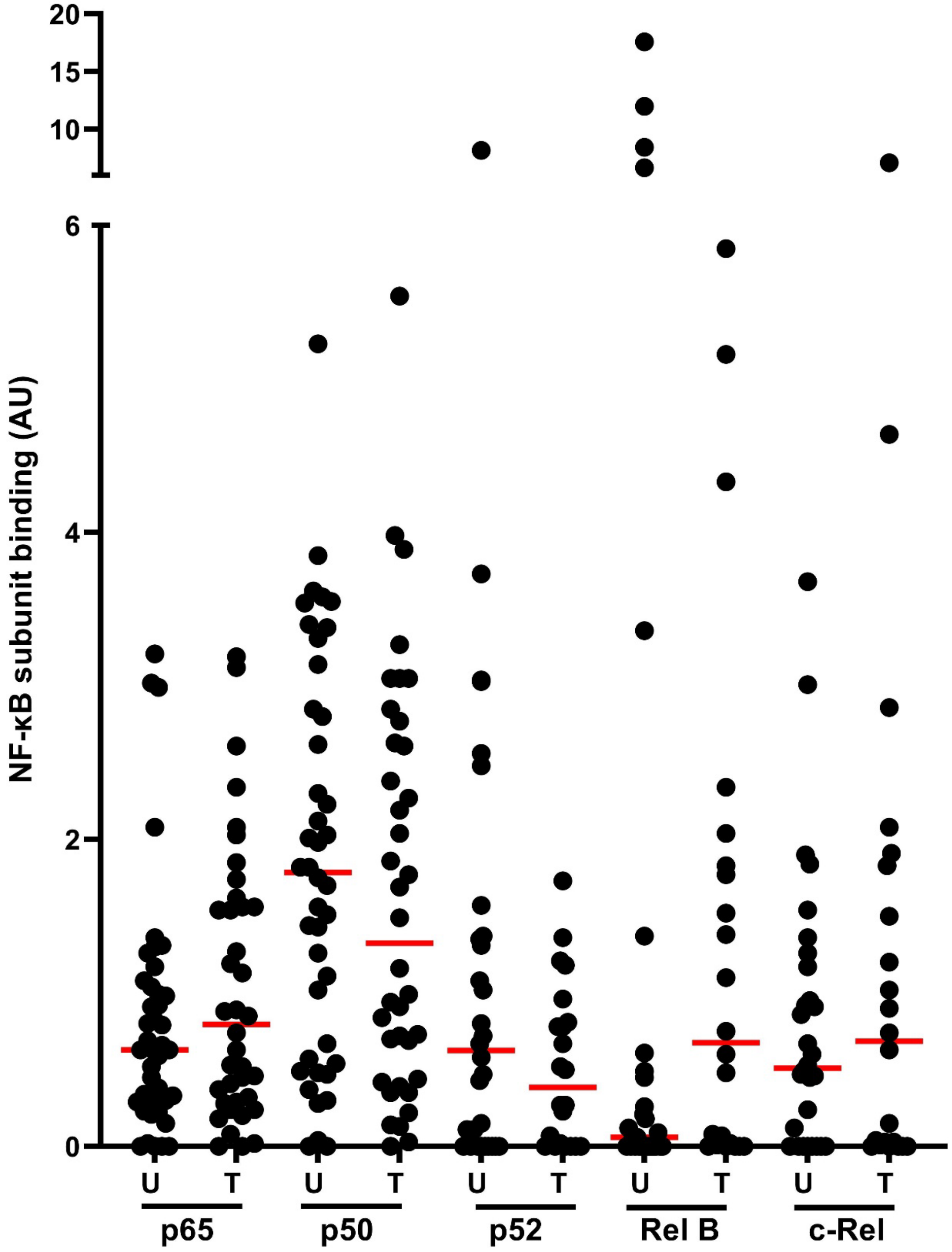

Supplementary Figure 1. **NF-kB subunit levels in treatment-naïve *versus* treated cases.** The scattergram shows DNA binding levels of NF-κB p65, p50, p52, c-rel and RelB subunits (as measured by ELISA) grouped into either group 'U' (untreated, i.e. treatment-naïve) or group 'T' for those that had received treatment at the time of sample collection. When analysed by unpaired T test (Mann Whitney) there was no significant difference in levels between treated and untreated for any of the subunits (p65, $p = 0.29$; p50, $p = 0.42$; p52, $p = 0.36$; Rel B, $p = 0.1$ and c-Rel, $p = 0.65$).

Original, uncropped western blot membranes

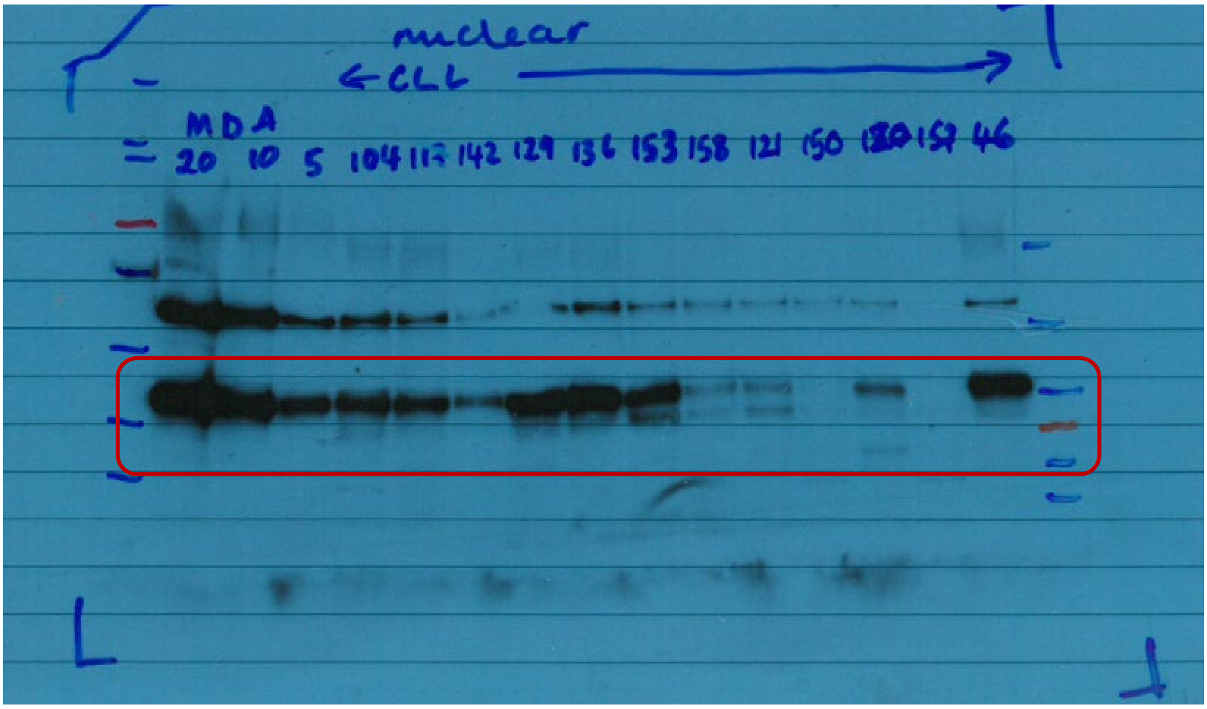

Figure 1B, RelB

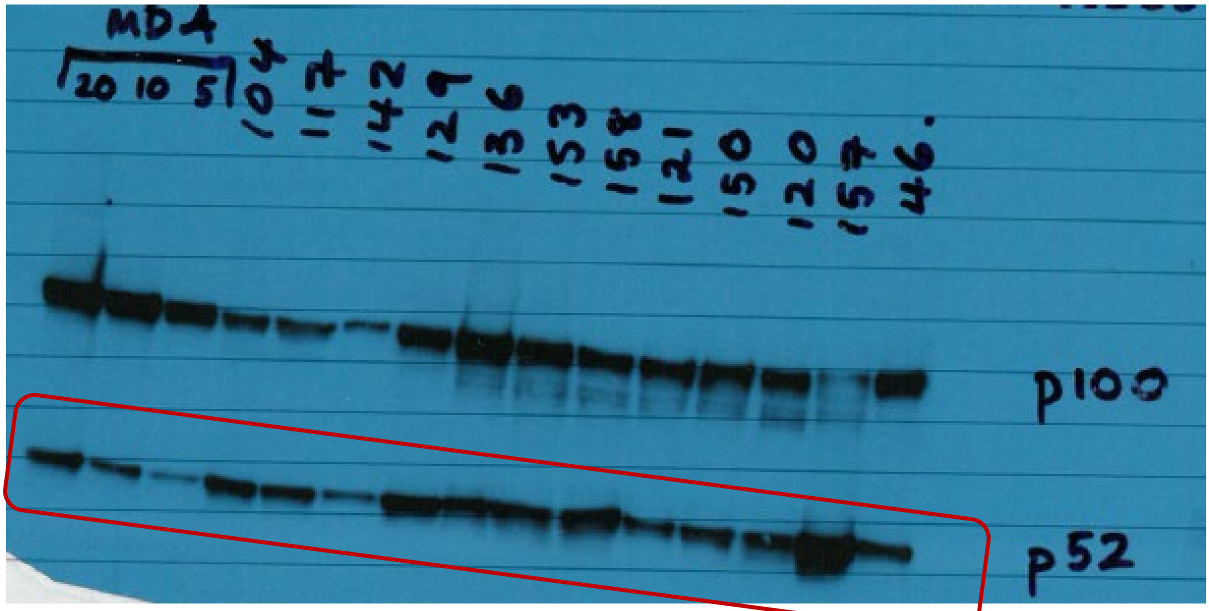

Figure 1B, p52

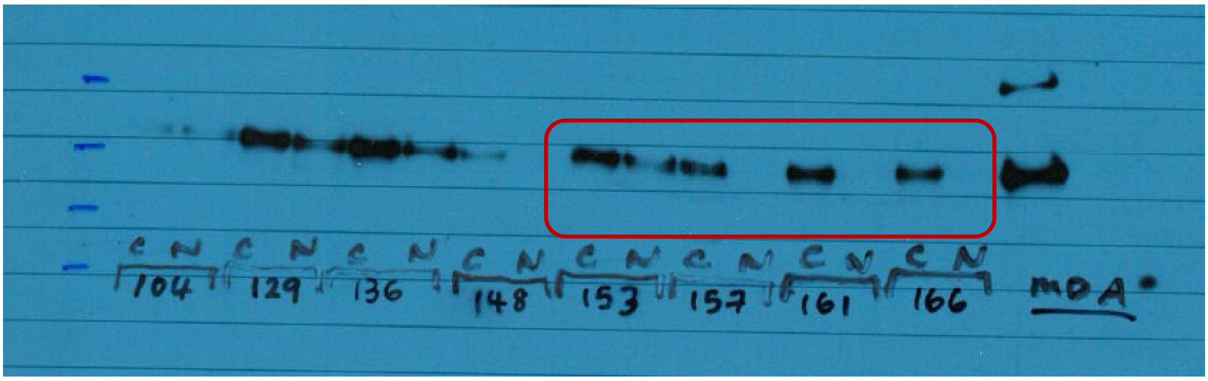

Figure 1D , RelB

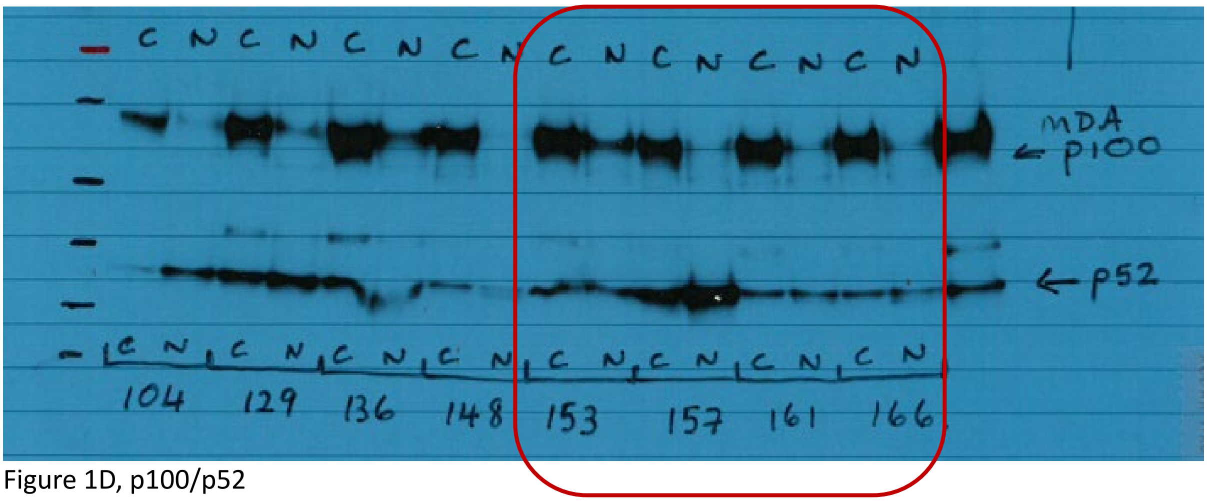

Figure 1D, p100/p52

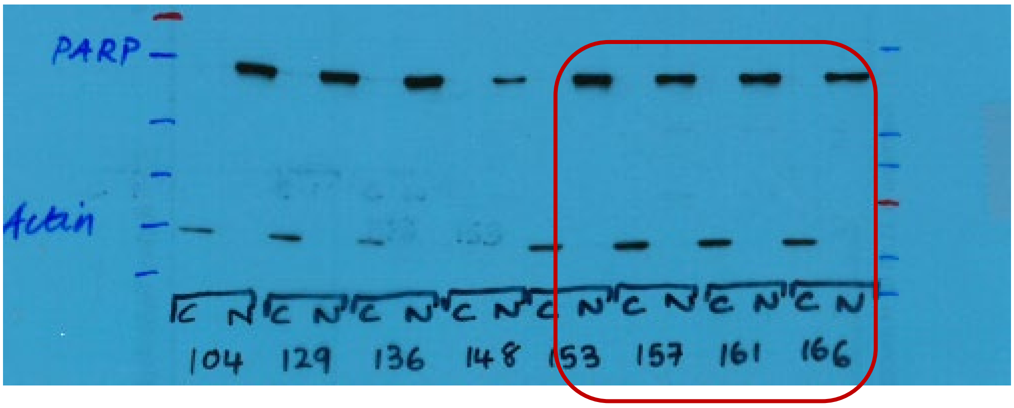

Figure 1D, PARP & Actin

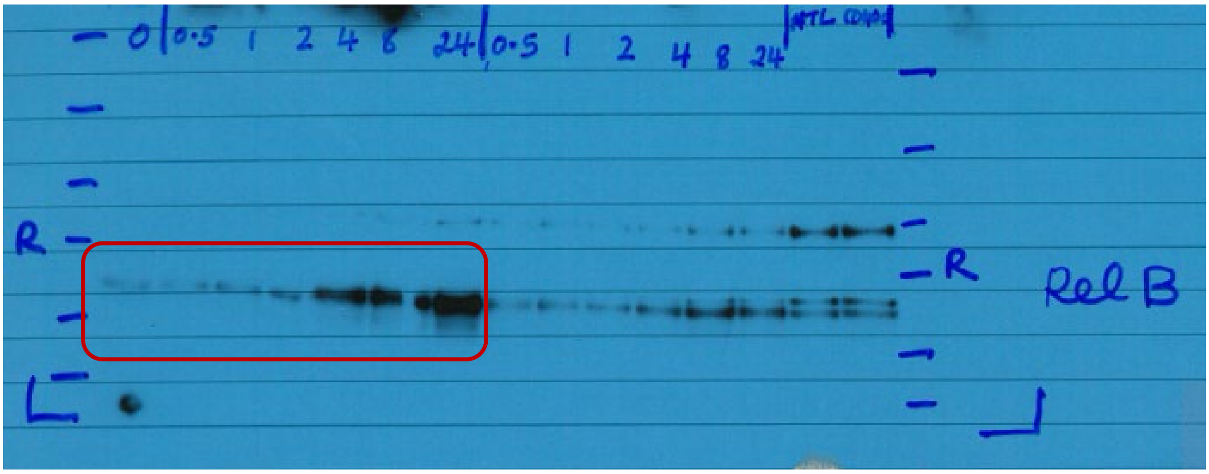

Figure 5A, (left hand side) RelB

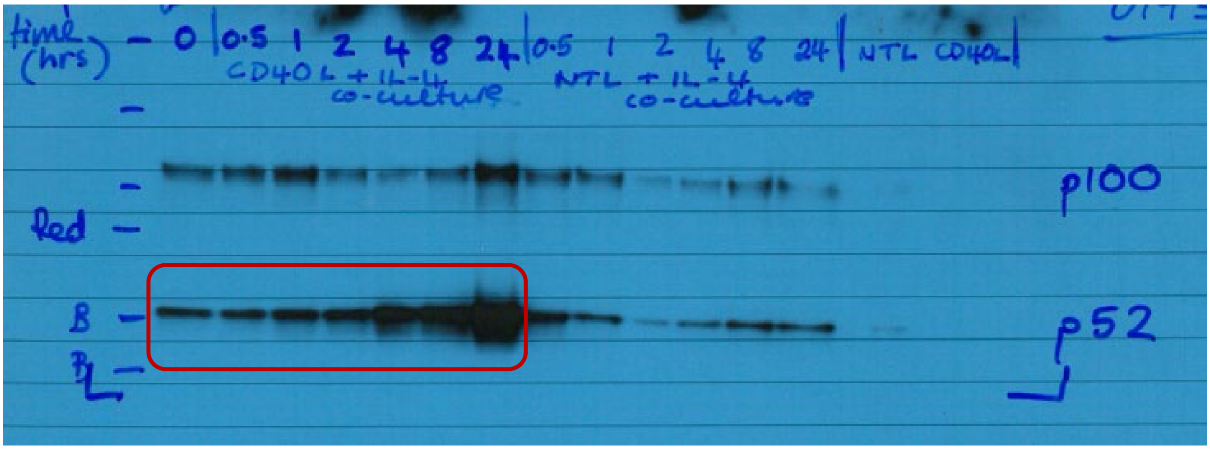

Figure 5A, (left hand side) p52

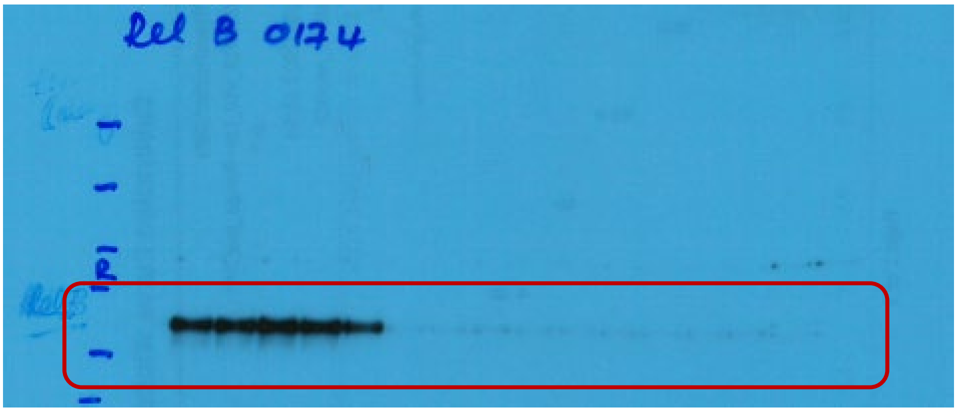

Figure 5A (right hand side) RelB

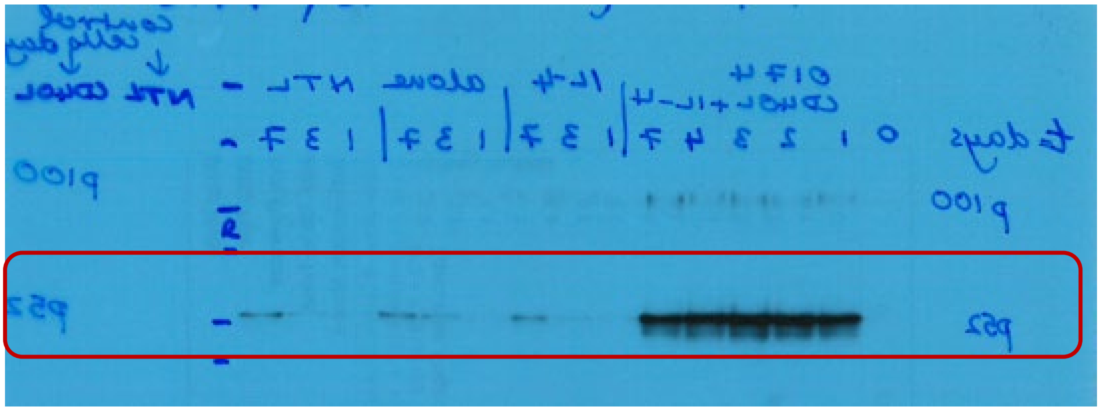

Figure 5A (right hand side, in reverse order) p52

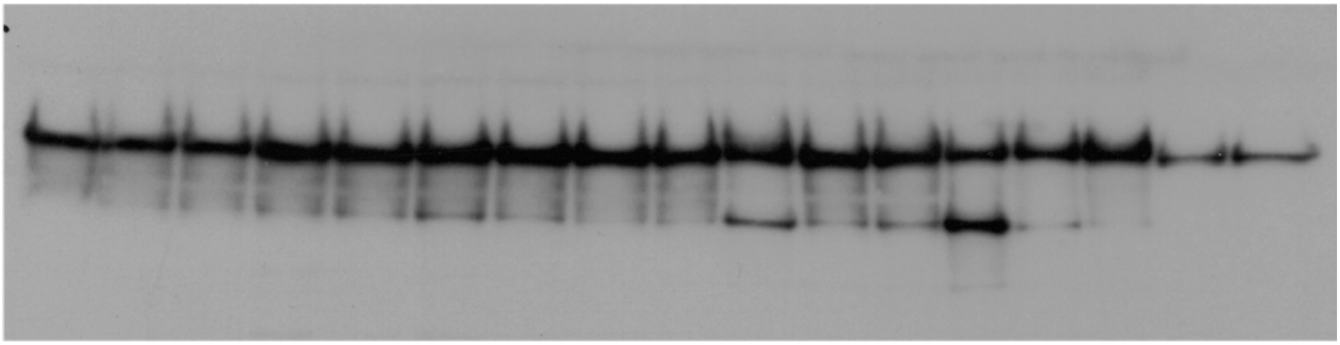

Figure 5A (right hand side) PARP

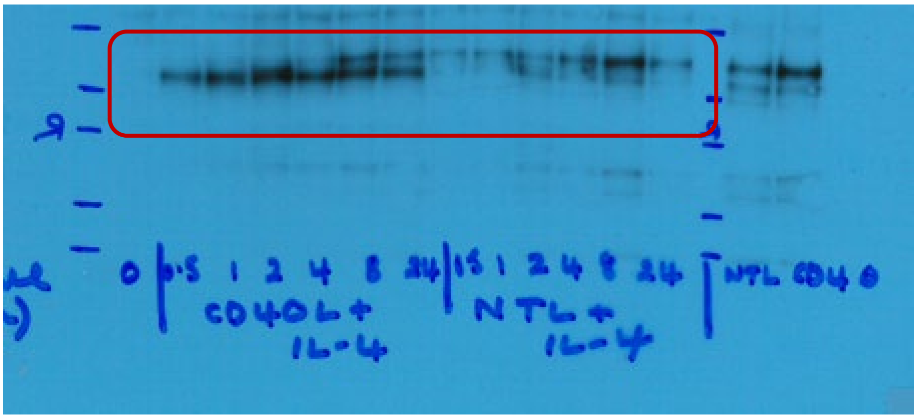

Figure 5b phospho p100

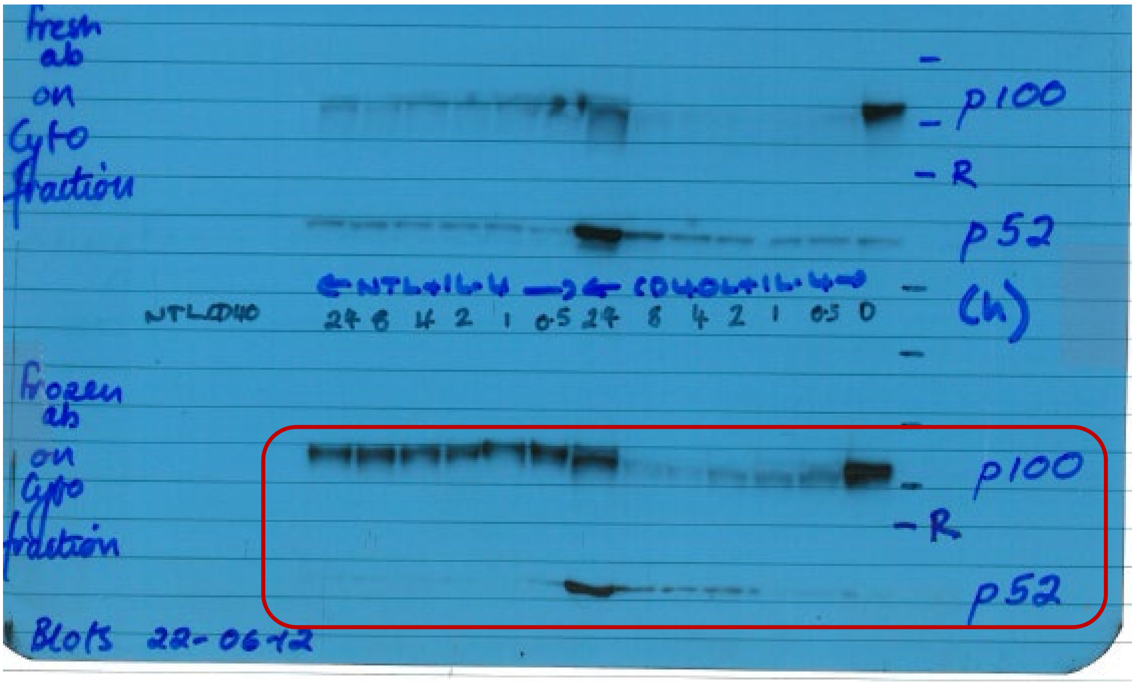

Figure 5b p100/p52
